# Nonscarring scalp alopecia: Which laboratory analysis should we perform on whom?

**DOI:** 10.3906/sag-2106-28

**Published:** 2021-10-23

**Authors:** Ümran ÖNER, Necmettin AKDENİZ

**Affiliations:** 1 Department of Dermatology, Erzurum Regional Training and Research Hospital, Health Science University, Erzurum, Turkey; 2 Department of Dermatology, Memorial Ataşehir Hastanesi, İstanbul, Turkey

**Keywords:** Alopecia areata, androgenetic alopecia, anemia, ferritin, telogen effluvium, vitamin D

## Abstract

**Background/aim:**

Vitamins and minerals are thought to play an essential but not entirely clear role in developing, preventing, and treating nonscarring alopecia. Telogen effluvium, androgenetic alopecia, and alopecia areata are the most common forms of nonscarring alopecias. We would like to present a different perspective on laboratory abnormalities in patients with nonscarring alopecia.

**Materials and methods:**

A total of 467 patients (287 females, 180 males) were included retrospectively: One hundred and sixty patients in the telogen effluvium group, 101 patients in the androgenetic alopecia group, 99 patients in the alopecia areata group, and 107 patients in the hair loss group (patients who could not be diagnosed with any nonscarring alopecia and wanted to have an analysis due to the complaint of hair loss). Sociodemographic data, diagnostic distribution, and laboratory findings (hemoglobin, ferritin, vitamin B12, vitamin D, and TSH) were evaluated and compared.

**Results:**

The most common diagnosis was telogen effluvium in females and androgenetic alopecia in males. In women, hemoglobin (12.2% vs. 1.1%) and ferritin deficiencies (22.3% vs. 8.9%) were significantly higher than in men (p < 0.001, p < 0.001) Ferritin, hemoglobin, and vitamin B12 levels were significantly lower, and the number of patients with vitamin D, ferritin, hemoglobin and vitamin B12 deficiencies were significantly higher in the telogen effluvium group compared to the other groups. Laboratory abnormalities were detected least in the hair loss group.

**Conclusion:**

The number of patients with the highest abnormalities in all parameters was observed in the telogen effluvium group and females; therefore, we mainly suggest examining female patients with telogen effluvium first. We may prefer not to immediately investigate the laboratory abnormalities and follow up patients with some treatments in the hair loss group.

## 1. Introduction

Hair loss is a widespread problem that concerns individuals of all ages. Patients who apply to dermatology outpatient clinics with this complaint constitute a significant part of our daily practice and are examined under two main headings, mainly nonscarring and scarring alopecia. Telogen effluvium (TE), androgenetic alopecia (AGA), and alopecia areata (AA) are the most common forms of nonscarring alopecias [1]. There are studies with conflicting results on the role of vitamin and mineral deficiencies, anemia, and thyroid dysfunction in nonscarring alopecia [2–7]. There is a general view that hair loss can be caused by vitamin deficiency, and patients who apply with hair loss complaints demand that vitamin values be investigated.

In this study, demographic data, diagnostic distribution, and laboratory parameters of patients diagnosed with nonscarring alopecia were evaluated. Patients were grouped according to diagnosis and statistically compared. We aimed to provide solutions to the question of “Which laboratory analysis should we perform at first on whom in patients with nonscarring alopecia?”

## 2. Materials and methods

A retrospective study was planned to evaluate the patients admitted with nonscarring hair loss complaints or diagnosed with nonscarring alopecia. We enrolled 467 patients from dermatology outpatient clinics of Mareşal Çakmak State Hospital and Regional Training and Research Hospital, Erzurum, Turkey, between November 2018 and January 2021. All patients were evaluated and diagnosed by the same dermatologist (Ü.Ö.). The study was approved by the ethics committee of Regional Training and Research Hospital, Erzurum, Turkey (decision no: 2021/01-12).

Age, sex, primary diagnoses, and laboratory findings were obtained from the electronic registration database. One hundred seven patients who cannot be diagnosed with any form of nonscarring alopecia and wanted to have an analysis due to hair loss complaints were included in the Hair Loss (HL) group. Moreover, 360 patients diagnosed with nonscarring alopecia (160 TE, 101 AGA, and 99 AA) were involved in the study. Levels of vitamin D (kit catalog no: 10995720), hemoglobin (Lysercell WDF® solution kit catalog no: AL-337-564), ferritin (kit catalog no: 10995568), vitamin B12 (kit catalog no: 10995715), and TSH (kit catalog no: 10995704) were measured by Atellica Solution Immunoassay, Siemens, USA.

The patients were diagnosed with TE, AGA, and AA based on the data obtained from anamnesis, dermatological, and trichoscopic examination. Since TE can usually be triggered by an etiological factor, systemic disease, fever, surgeries, medications, diet, postpartum period, and stress were questioned. In dermatological examination, a hair-pulling test, which is an important clue for particularly TE, was performed for each patient. Also, trichoscopic findings were evaluated. Hair diameter diversity, peripilar sign (perifollicular pigmentation), and yellow dots are trichoscopic features of AGA. In AA, black dots, tapering hairs, broken hairs, yellow dots, and short vellus hairs are observed, and black dots, tapering hairs, and broken hairs are considered as pathognomonic findings [8].

Reference ranges of hematinic parameters were accepted as follows: hemoglobin (M: 13–17.8 g/dL, F: 12.2–15.9 g/dL), ferritin (M: 30–400 ng/mL, F: 11–150 ng/mL), vitamin B12 (197–771 ng/mL), vitamin D (20–70 ng/mL), TSH (0.27–4.20 uUI/mL). Patients with abnormal TSH levels (<0.27,> 4.20 uUI/mL) were investigated for free thyroxine (FT4), free triiodothyronine (FT3) and then the decision was made for hypotrioidism or hyperthyroidism.

Statistical Package for Social Sciences (SPSS Inc, Chicago, IL, USA) for Windows 22.0 program was used for statistical analysis. Chi-square test was used to compare categorical data. Chi-square test was used to compare groups according to sex and hematinic parameters, as well as to compare sexes according to hematinic parameters. After the chi-square analysis, paired comparisons were made, and Bonferroni correction was made to determine the group from which the difference originated. The suitability of the parameters to the normal distribution was evaluated with the Kolmogorov-Smirnov test. While evaluating the study data, in addition to descriptive statistical methods (mean, standard deviation, number, and percentage), the One-Way ANOVA test was used for comparisons of parameters normally distributed in quantitative data. One Way ANOVA test was used to compare the groups in terms of age and hematinic parameters. Post-hoc test (Tukey test) was used to find out where significance originated. The value of p < 0.05 was considered statistically significant.

## 3. Results

A total of 467 individuals, 287 females (61.4%) and 180 males (38.6%), applied with the complaint of hair loss between the ages of 2–58 years and were examined due to nonscarring alopecia were included in our study retrospectively. One hundred and sixty patients (34.2%) were diagnosed with TE, 101 patients (21.6%) AGA, 99 patients (21.1%) AA, and 107 patients (22.9%) as HL. While TE and HL diagnoses were higher in females, the male sex was more dominant in AGA and AA groups. The age and sex distribution of the groups is shown in Table 1.

Vitamin D deficiency in 345 patients (73.8%), hemoglobin deficiency in 37 patients (7.9%), ferritin deficiency in 80 patients (17.1%), vitamin B12 deficiency in 54 patients (11.5%), thyroid dysfunction in 31 patients (6.6%) were detected. At least a hematinic deficiency was present in 143 patients (89.4%) in the TE group, 84 patients (83.2%) in the AGA group, 77 patients (77.8%) in the AA group, and 70 patients (65.4%) in the HL group.

The deficiency of at least one of the hematinic values was found as 81.5% in women and 77.8% in men. The most common deficiency was observed in both sexes was vitamin D deficiency (Table 2). The second was ferritin deficiency in women and vitamin B12 deficiency in men. In women, hemoglobin deficiency (12.2% vs. 1.1%) and ferritin deficiency (22.3% vs. 8.9%) were significantly higher than in men (p < 0.001, p < 0.001, Table 2).

Mean values of hematinic parameters were given in Table 3, and a comparison of groups according to the number of patients with hematinic deficiencies was given in Table 4.

### 3.1. Vitamin D

The mean vitamin D levels in all groups were found below the reference range: TE (14.6 ± 11.1 ng/mL), AGA (16.2 ± 8.4 ng/mL), AA (15.7 ± 9.6 ng/mL), HL (17, 9 ± 11.3 ng/mL) (p= 0.086, Table 3). Vitamin D deficiency was detected in 127 patients (79.4%) in the TE group, 79 patients (78.2%) in the AGA group, 70 patients (70.7%) in the AA group, 69 patients (64.5%) in the HL group (Table 4, Figure). The number of patients with vitamin D deficiency was significantly higher in the TE group (p= 0.031, Table 4).

### 3.2. Hemoglobin

The mean hemoglobin of the groups was 13.5 ± 1.6 g/dL in the TE group, 16.1 ± 1.4 g/dL in the AGA group, 15.4 ± 1.54 g/dL in the AA group, 14.8 ± 1.4 g/dL in the HL group (p < 0.001, Table 3). Hemoglobin deficiency was detected in 32 patients (20%) in the TE group, four patients (4%) in the AA group, and one patient (0.9%) in the HL group (Table 4). No hemoglobin deficiency was found in the patients with AGA (Figure). The mean hemoglobin in the TE group was significantly lower, and the number of patients with hemoglobin deficiency was significantly higher than the other groups (p < 0.001, p < 0.001, Table 3,4).

### 3.3. Ferritin

The mean ferritin values were 28.3 ± 37.4 ng/mL in the TE group, 100.0 ± 84.7 ng/mL in the AGA group, 93.5 ± 103.9 ng/mL in the AA group, and 59.3 ± 47.6 ng/mL in the HL group (p < 0.001, Table 3). Ferritin deficiency was detected in 53 patients (33.1%) of the TE group, eight patients (7.9%) of the AGA group, 13 patients (13.1%) of the AA group, and six patients (5.6%) of the HL group (Table 4, Figure). Ferritin levels were significantly lower in the TE group than the other groups, and the number of patients with ferritin deficiency was significantly higher (p < 0.001, p < 0.001, Table 3.4).

### 3.4. Vitamin B12

The mean vitamin B12 levels in all groups were in the reference range given as follows: TE (317.7 ± 134.4 pg/mL), AGA (316.8 ± 170.5 pg/mL), AA (344.6 ± 150.4 pg/mL), HL (377.1 ± 154.9 pg/mL) (p= 0.007, Table 3). Vitamin B12 deficiency was detected in 32 patients (20%) in the TE group, 11 patients (10.9%) in the AGA group, eight patients (8.1%) in the AA group, and three patients (2.8%) in the HL group (Figure). Mean vitamin B12 levels were significantly lower in the TE and AGA groups compared to the AA and HL groups, while the number of patients with vitamin B12 deficiency was significantly higher only in the TE group compared to the other groups (p= 0.007, p < 0.001, Table 3,4).

### 3.5. Thyroid Stimulating Hormone

The mean of TSH levels were 2.2 ± 1.6 UI/mL in the TE group, 2.1 ± 1.4 UI/mL in the AGA group, 2.3 ± 2.0 UI/mL in the AA group, 2.2 ± 1.1 UI/mL in the HL group (p= 0.943, Table 3). Twelve patients in the TE group had hypothyroidism, and three patients had hyperthyroidism. Six patients in the AGA group had hypothyroidism, and none of the patients had hyperthyroidism. Seven patients in the AA group had hypothyroidism, and one patient had hyperthyroidism. Three patients in the HL group had only hypothyroidism. There was no significant difference between the groups in thyroid dysfunction (Table 4).

## 4. Discusson

Vitamin-mineral deficiencies and laboratory abnormalities are often mentioned in nonscarring alopecia, and there is still no consensus on the exact date. Vitamins and minerals are thought to play an essential but not entirely clear role in the hair follicle cycle, and their deficiencies may be associated with a modifiable risk factor in the development, prevention, and treatment of alopecia [1]. In our study, patients presented with nonscarring hair loss were grouped according to diagnosis (TE, AGA, AA, HL), and demographic data, and laboratory parameters (hemoglobin, ferritin, vitamin B12, vitamin D, thyroid function) were evaluated in each group. The patients were diagnosed with TE, HL, AGA, and AA in order of frequency. The most common diagnosis was TE in females and AGA in males. It was observed that the female/male ratio of patients was 1.5/1, and the mean age was in the 3rd decade in all groups. In the HL group, patients under 30 (%85) were the most common. Laboratory abnormalities were detected least in the HL group and the most in the TE group.

A recent review suggested that iron deficiency is more common in female patients with non-scarring alopecia, iron supplementation in TE or AGA, and vitamin D supplementation in TE, AGA, and AA may be beneficial. However, they suggested insufficient evidence for vitamin B12 supplementation in TE, AGA, or AA [1]. In our study, the mean vitamin D levels in all groups were below the reference range, and the most common deficiency was vitamin D deficiency (73.8%). After administering vitamin D to mice with congenital alopecia, Vegesna et al. observed that hair growth was stimulated dramatically, and expression of certain keratins (Ha7, Ha8, and Hb3) increased. They stated that vitamin D might affect keratinocytes to stimulate hair growth and initiate the hair follicle cycle [9]. It has also been reported that as vitamin D levels decreased in TE, AGA, and AA, the severity of the disease increased [3,10]. On the contrary, some authors reported that the vitamin D levels of patients with AA and the control group were similar, and there was no relationship between AA and vitamin D [11].

In a study evaluating vitamin D levels and anemia, vitamin D levels were significantly higher in patients with TE than controls, and it was suggested that this might be associated with increased exposure to ultraviolet light due to TE. Ferritin and hemoglobin levels were significantly lower in patients, and the authors pointed out that iron deficiency anemia may be the main trigger factor in TE [12]. In our study, the most common deficiency after vitamin D deficiency was ferritin deficiency. Iron deficiency has often been associated with nonscarring alopecia, particularly TE. Serum ferritin (<41 ng/mL) is considered one of the most sensitive and specific markers for iron deficiency [13].^1^ The ferritin level to employ in patients with hair loss has not been determined yet, but 70 μg/L, with a normal erythrocyte sedimentation rate (<10 mm/h), has been recommended [6]. In our study, the mean ferritin was lower than 40 ng/mL in only the TE group. The rate of patients with ferritin level <40 ng/mL was 126/160, and the rate of patients with ferritin level <70 ng/mL was 150/160 in the TE group. In both cases where the cut-off level was 40 or 70 ng/mL, ferritin deficiency was most observed in the TE group.

However, some studies proposed different results, such as low ferritin level, associated with dense hair loss rather than mild hair loss or no relationship between ferritin deficiency and alopecia [4,14,15]. Kantor et al. found serum ferritin significantly lower in AGA and AA than the control group but not in TE [14]. However, in this study, the mean age of the TE group was 47.9 and higher than other groups. Postmenopausal female patients were more likely to be in the TE group due to the higher mean age, and this probability may be the reason for the high ferritin levels [15]. In a study similar to our results, it was reported that iron deficiency anemia could not be detected in any male and female patients diagnosed with AGA [2]. In our study, low hemoglobin was detected in none of the patients with AGA, the mean ferritin was found higher than 70 ng/mL, and the number of patients with ferritin deficiency (8/101) was quite low in the AGA group. In conclusion, we suggest that AGA may not be associated with iron deficiency and anemia.

As we compared our results with a retrospective study conducted with AA patients, the male/female ratio in our study was higher (2.9 vs. 1.8), and the mean age was younger (26.1 vs. 29.6). Hemoglobin deficiency (4% vs. 11.1%) and thyroid dysfunction (8% vs. 10%) were less common in our study, while ferritin (13.1% vs. 10.6%) and vitamin B12 deficiencies (8.1% vs. 3.9%) were more common [5]. Our results suggested that AA may be associated with ferritin deficiency rather than anemia, but a study, comparing ferritin, iron, and iron-binding capacities between AA patients and controls, reported no association between iron deficiency and AA [16].

Vitamin B12 is suggested to have a role in hair follicle proliferation, as it is essential for DNA synthesis [17–18].^2^ However, there is still no sufficient data to recommend supplementation of vitamin B12 in TE, AGA, and AA [1]. In a case-control study examining vitamin B12 deficiency in women with TE, there was no difference between the groups in mean vitamin B12 levels, while the number of patients with vitamin B12 deficiency (11.9% vs. 6.7%) was significantly higher than controls [17]. In our study, mean vitamin B12 levels were not below the reference range in any group, and the highest number of patients with vitamin B12 deficiency was in the TE group (%20). Contrary to our results, in a study conducted with 563 TE patients, the number of patients with vitamin B12 deficiency (3%) was found to be quite low [19]. In a case-control study, Gönül et al. found no significant difference in vitamin B12 levels between AA patients and controls; thus, they reported that vitamin B12 does not play a role in the etiology and activity of AA [20]. However, the number of individuals with vitamin B12 deficiency was not presented in the report and can be a limitation of this study.

Thyroid hormones are necessary for the normal cycle of hair and normal sebum secretion [21]. Hale and Ebling showed the effect of thyroid hormone on the hair growth cycle in rats given intraperitoneal Thyroxine (T4) and reported that T4 decreased both the anagen phase and the telogen phase [22]. In addition, the T3 hormone has been shown to stimulate the proliferation or metabolism of outer root sheath keratinocytes, dermal papilla cells, and sheath cells of hair follicles [23]. Diffuse non-cicatricial alopecia, loss of pigment in the hair, and premature graying may occur in individuals with thyroid dysfunction [24,25]. The highest number of patients with thyroid dysfunction was in the TE group (9.4%), and the second was in the AA group (8.1%) in our study. Already, in the literature, thyroid dysfunction has been mostly associated with TE and AA. In a study conducted with 151 TE patients, thyroid dysfunction was found in 23 patients (15.2%), and it was suggested that thyroid dysfunction might be related to TE [7]. A recent meta-analysis stated that the prevalence of thyroid diseases in AA patients was significantly higher than in controls, and it was recommended that AA patients be examined for thyroid diseases [26]. It is suggested to investigate thyroid autoantibodies to determine those with a risk of thyroid disease, even if patients with AA are euthyroid [25]. As a result, it should be helpful to examine patients with TE and AA for thyroid dysfunction.

In this study, the relationship between hair loss and laboratory abnormalities, which continues to be discussed and still has no definitive results, was examined. Solutions were offered to the question of which laboratory examination should be done on which patient. We may prefer not to investigate the laboratory abnormality immediately and follow up patients with the treatments given initially in young patients admitted due to hair loss but do not have the necessary criteria for any diagnosis of nonscarring alopecia (TE, AGA, or AA). The number of patients with the highest abnormalities in all parameters was observed in TE and females; therefore, we particularly suggest examining patients with TE and female patients first. More studies had been reported on laboratory abnormalities in TE, and they may also support our suggestion [12,17,17,27–29]. After vitamin D deficiency in both sexes, investigation ferritin and hemoglobin deficiency in females and vitamin B12 deficiency in males may be recommended. Limitations of our study were that a control group could not be formed as there was no homogeneous distribution in sociodemographic data in the groups, and it included only patients in a particular region retrospectively. Multicenter studies conducted with control groups in different regions will contribute more to revealing precise results.

## Figures and Tables

**Figure:**
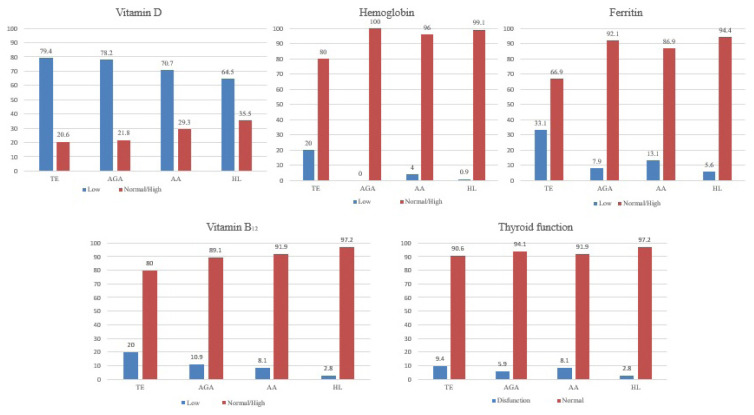
Low or normal/high ratios of hematinic parameters in all groups.

**Table 1:** Sociodemographic data of the groups.

	Age (years)	Age range (years)	Female		Male		Total
	*M ± SD*		n	%	n	%	n
**TE**	27.7 ± 8.8^a^	7–55	156	97.5^a^	4	2.5	160
**AGA**	25.6 ± 7.3^a^	15–50	25	24.8^b^	76	75.2	101
**AA**	26.1 ± 12.3^a^	2–58	25	25.3^b^	74	74.7	99
**HL**	21.4 ± 9.2^b^	2–55	81	75.7^c^	26	24.3	107
**Total**	25.5 ± 9.7	2–58	287	61.4	180	38.6	467
**p**	***<0.001*** *		***<0.001*** **				

M ± SD: Mean ± Standart deviation, n: Number,

* One Way ANOVA test,

** Chi-square test (comparison of groups according to sex),

a,b,c Different letters indicate the statistical difference.

**Table 2:** Comparison of hematinic deficiencies and thyroid dysfunction in all patients according to sex.

	Vitamin D deficiency		Hemoglobin deficiency		Ferritin deficiency		Vitamin B_12_ deficiency		Thyroid dysfunction	
	*n*	*%*	*n*	*%*	n	%	n	%	n	%
Female	215	74.9	35	12.2	64	22.3	33	11.5	22	7.7
**Male**	130	72.2	2	1.1	16	8.9	21	11.7	10	5.6
**p** *	0.519		***<0.001***		***<0.001***		0.956		0.380	

* Chi square test, n: Number

**Table 3:** Comparison of hematinic parameters in groups.

	Vitamin D (ng/mL)	Hemoglobin (g/dL)	Ferritin (ng/mL)	Vitamin B_12_ (pg/mL)	TSH (UI/mL)
	M ± SD	M ± SD	M ± SD	M ± SD	M ± SD
**TE**	14.6 ± 11.1	*13.5 ± 1.6* a	*28.3 ± 37.4* a	*317.7 ± 134.4* a	2.2 ± 1.6
**AGA**	16.2 ± 8.4	16.1 ± 1.4^b^	100.0 ± 84.7^b^	*316.8 ± 170.5* a	2.1 ± 1.4
**AA**	15.7 ± 9.6	15.4 ± 1.5^c^	93.5 ± 103.9^b^	344.6 ± 150.4a,b	2.3 ± 2.0
**HL**	17.9 ± 11.3	14.8 ± 1.4^d^	59.3 ± 47.6^c^	377.1 ± 154.9^b^	2.2 ± 1.1
**p** *	0.086	***<0.001***	***<0.001***	***0.007***	0.943

* One Way ANOVA test,

a,b,c,d Different letters indicate the statistical difference,

M ± SD: Mean ± Standart deviation.

**Table 4:** Comparison of the groups according to hematinic deficiencies and thyroid dysfunction.

	Vitamin D deficiency		Hemoglobin deficiency		Ferritin deficiency		Vitamin B_12_ deficiency		Thyroid dysfunction	
	n	%	n	%	**n**	**%**	**n**	**%**	**n**	**%**
**TE**	*127*	*79.4* a	*32*	*20.0* a	*53*	*33.1* a	*32*	*20.0* a	15	9.4
**AGA**	*79*	*78.2* a,b	0	0.0^b^	8	7.9^b^	11	10.9a,b	6	5.9
**AA**	*70*	*70.7* a,b	4	4.0^b^	13	13.1^b^	8	8.1a,b	8	8.1
**HL**	69	64.5^b^	1	0.9^b^	6 b	5.6^b^	3	2.8^b^	3	2.8
**p** *	*0.031*		***<0.001***		***<0.001***		***<0.001***		0.194	

* Chi square test,

a,b Different letters indicate the statistical difference,

n: Number

## Footnotes

1 Schrier SL (2008). Causes and diagnosis of anemia due to iron deficiency [online]. Website: http://www.uptodate.com. [accessed September 2008].

2 Harvard TH Chan, School of Public Health (2018). Three of the B vitamins: folate, vitamin B6, and vitamin B12[online]. Website https://www.hsph.harvard.edu/nutritionsource/vitamins/vitamin-b/ [Accessed 8 Aug 2018]
